# Impact of an online writing aid tool for writing a randomized trial report: the COBWEB (Consort-based WEB tool) randomized controlled trial

**DOI:** 10.1186/s12916-015-0460-y

**Published:** 2015-09-15

**Authors:** Caroline Barnes, Isabelle Boutron, Bruno Giraudeau, Raphael Porcher, Douglas G Altman, Philippe Ravaud

**Affiliations:** Centre d’Épidémiologie Clinique, Hôpital Hôtel Dieu, Assistance Publique des Hôpitaux de Paris, Hôpital Hôtel Dieu, Aile A2 1er étage 1, Place du parvis Notre Dame, 75181 Paris, Cedex 4, France; Paris Descartes University, Paris, France; INSERM, UMR 1153, Centre of Research in Epidemiology and Statistics Sorbonne Paris Cité – (CRESS), METHODS team, Paris, France; INSERM CIC 1415, Université François-Rabelais de Tours; CHRU de Tours, Tours, France; Centre for Statistics in Medicine, Nuffield Department of Orthopaedics, Rheumatology & Musculoskeletal Sciences, University of Oxford, Oxford, UK; Department of Epidemiology, Columbia University Mailman School of Public Health, New York, NY USA

**Keywords:** Clinical epidemiology, CONSORT statement, Randomized controlled trial, Reporting guidelines, Transparency

## Abstract

**Background:**

Incomplete reporting is a frequent waste in research. Our aim was to evaluate the impact of a writing aid tool (WAT) based on the CONSORT statement and its extension for non-pharmacologic treatments on the completeness of reporting of randomized controlled trials (RCTs).

**Methods:**

We performed a ‘split-manuscript’ RCT with blinded outcome assessment. Participants were masters and doctoral students in public health. They were asked to write, over a 4-hour period, the methods section of a manuscript based on a real RCT protocol, with a different protocol provided to each participant. Methods sections were divided into six different domains: ‘trial design’, ‘randomization’, ‘blinding’, ‘participants’, ‘interventions’, and ‘outcomes’. Participants had to draft all six domains with access to the WAT for a random three of six domains. The random sequence was computer-generated and concealed. For each domain, the WAT comprised reminders of the corresponding CONSORT item(s), bullet points detailing all the key elements to be reported, and examples of good reporting. The control intervention consisted of no reminders. The primary outcome was the mean global score for completeness of reporting (scale 0–10) for all domains written with or without the WAT.

**Results:**

Forty-one participants wrote 41 different manuscripts of RCT methods sections, corresponding to 246 domains (six for each of the 41 protocols). All domains were analyzed. For the primary outcome, the mean (SD) global score for completeness of reporting was higher with than without use of the WAT: 7.1 (1.2) versus 5.0 (1.6), with a mean (95 % CI) difference 2.1 (1.5–2.7; *P* <0.01). Completeness of reporting was significantly higher with the WAT for all domains except for blinding and outcomes.

**Conclusion:**

Use of the WAT could improve the completeness of manuscripts reporting the results of RCTs.

**Trial registration:**

Clinicaltrials.gov (http://clinicaltrials.govNCT02127567, registration date first received April 29, 2014)

**Electronic supplementary material:**

The online version of this article (doi:10.1186/s12916-015-0460-y) contains supplementary material, which is available to authorized users.

## Background

Inadequate reporting is a frequent cause of waste in research [1, 2]. To overcome this issue, the CONSORT Statement [3], an evidence-based, minimum set of recommendations for reporting randomized controlled trials (RCTs), was developed, along with extensions for reporting specific designs (e.g. cluster [4], non-inferiority [5]), data (e.g. harms [6]), and interventions (non-pharmacologic treatments [7]). The CONSORT guidelines have received support from the World Association of Medical Editors, the Council of Science Editors, and the International Committee of Medical Journal Editors. According to the CONSORT website [8], currently 585 journals worldwide endorse CONSORT. Most journals include recommendations to follow the main CONSORT statement in their instructions to authors and some have specific policies to enforce the use of the CONSORT guidelines at the submission or revision stage of a manuscript [9].

However, the adherence of authors to these guidelines is low and the quality of reporting remains insufficient [9–21]. For example, the method of randomization was described in only 25 % of published RCTs [10, 15, 22], and more than 30 % of reports did not provide sufficient details to allow replication of the treatment evaluated in the trial in clinical practice [11, 23].

Several barriers can explain the lack of adherence to the CONSORT guidelines [1, 24–27]. Our hypothesis is that the strategy currently used to implement the reporting guidelines, such as the CONSORT Statement and its extensions, could be improved. First, the timing of implementation of CONSORT is late in the process. In fact, most active strategies used to improve the quality of reporting are implemented by editors and consist of asking authors to submit the CONSORT checklist at the submission or acceptance stage [9, 20]. Modifying a manuscript ready for submission is probably more difficult than writing the first draft of the manuscript according to the guidelines. Implementation at an earlier stage when writing the manuscript, instead of the submission or revision stages, could be more efficient.

Second, the CONSORT Statement and its extension are disseminated in two formats: (1) the CONSORT Statement (a checklist and flow diagram) and (2) the CONSORT “Explanation and Elaboration” document [3, 7]. The checklist is probably not sufficient for some authors to adequately understand what should be reported. For example, to report the item dedicated to the intervention “*Description of the different components of the interventions* [..]” authors reporting a surgical trial may benefit from more support to understand that they should report, in addition to the surgical procedure, all of the following information: details on preoperative care, postoperative care, and anesthesia management. The Explanation and Elaboration documents are meant to help authors understand the checklist. However, these documents are very long (more than 30 pages) and they combine explanations about why the item should be reported, how the item is reported in the literature, what should be reported, and examples of adequate reporting. Consequently, the important information is buried in the manuscript. The use of a template shell with a clear and explicit reminder of what should be reported when authors are writing their manuscript could be useful to increase adherence to the guidelines.

Finally, authors may need more support to adequately combine the different extensions of the CONSORT statement. In fact, to adequately report a cluster RCT evaluating a rehabilitation program, authors must rely on three checklists and three Elaboration and Explanation documents for the main CONSORT, the extension for cluster trials, and the extension for non-pharmacologic treatments. Up to now, no support has been developed to help authors successfully adhere to reporting guidelines at the writing stage of the manuscript.

An online writing aid tool that combines the different extensions of the CONSORT Statement and provides appropriate explanations with examples of adequate reporting would be a more efficient way to implement the CONSORT statements and thus improve the quality of reporting. We developed a writing aid tool based on the CONSORT guidelines and its extension for non-pharmacologic treatments to help authors when writing a report of a RCT. We evaluated the impact of this tool on the completeness of reporting of two-arm parallel-group RCTs evaluating pharmacologic and non-pharmacologic interventions.

## Methods

### Trial design

**Fig. 1:**
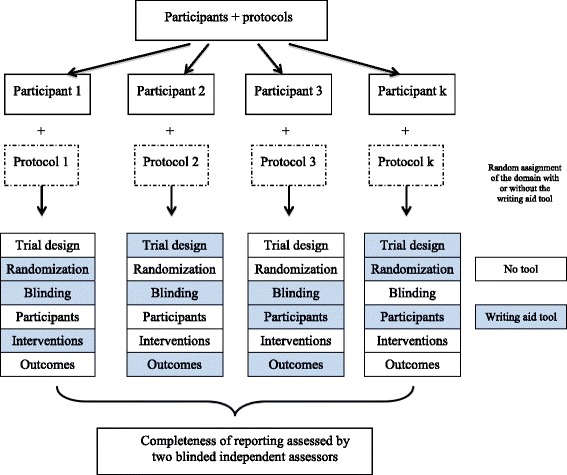
Schema of the study design

We performed a ‘split-manuscript’ RCT with blinded outcome assessment. The methods section of each manuscript was divided into six different domains: ‘trial design’, ‘randomization’, ‘blinding’, ‘participants’, ‘interventions’, and ‘outcomes’. Each participant was randomly allocated to a different real RCT protocol. Participants had to write the six domains of the methods section of the manuscript for the protocol they received over a 4-hour period. They had access to the tool for a random three of the six domains. Thus, the unit of randomization was the domain, embedded within the manuscript (Fig. 1).

Authorization by the CNIL (“Commission Nationale de l’Informatique et des Libertés”; file number 1753007) whose remit is to protect participants’ personal data and the institutional review board of INSERM ethics committee (IRB 00003888) was obtained and the study protocol was registered at ClinicalTrials.gov (http://clinicaltrials.gov NCT02127567).

Informed consent was obtained from all participants. The consent was obtained electronically. All parts of the trial were conducted in Paris.

### Randomization

#### Sequence generation

The randomization sequence was computer-generated with the use of SAS 9.2. For each participant-manuscript, three domains were allocated to the ‘writing aid tool’ group and the three remaining domains were allocated to the ‘usual writing’ group. Allowing for 20 possible combinations of domains (i.e. three of six domains with the tool and three without), randomization was performed with permuted blocks of 20.

#### Implementation

Only the independent statistician and the computer programmer who developed the online writing aid tool and the website had access to the randomization list. The statistician who generated the list (BG) provided the list to the programmer, who uploaded it on the study’s secure website. The list was not available to the researchers who enrolled the participants and were present at the various study sessions (CB, IB).

#### Allocation concealment

The sequence was concealed by a computer interface.

### Blinding

Participants could not be blinded to intervention assignments. However, outcome assessors were blinded to intervention assignments.

### Participants

Study participants were masters or doctoral students in the field of public health and medical research who were based in Paris and who were affiliated with Paris Descartes University, Pierre and Marie Curie University, and Paris Diderot University, or the Mailman School of Public Health of Columbia University, in New York.

An e-mail advertisement was sent to students to invite them to participate in a writing session. Participants were not informed of the study in the email advertisement. Before obtaining their consent, participants attended a small informational session with a PowerPoint presentation describing the writing task to perform. Participants were instructed to complete six sections of a manuscript describing the study protocol they were provided and that they would have assistance for three sections and no assistance for three sections, although they were not instructed as to which sections. They were informed that this design had a pedagogical purpose as they could see how useful it was to have the writing aid tool and use reporting guidelines when writing the first draft of a manuscript. They were instructed that we would use their results to evaluate the impact of the tool. Before beginning the exercise, participants provided their consent electronically.

### Selection of protocols

We retrieved all protocols of RCTs published between January 1, 2013, and March 28, 2014, in the *New England Journal of Medicine* or the *Journal of Clinical Oncology*. We chose these journals because they provide access to the protocol for all the RCTs they publish.

One researcher (CB) searched MEDLINE via PubMed (search strategy is reported in Additional file 1) and screened all titles and abstracts retrieved to select all reports of two-arm parallel-group RCTs. All available protocols published in English were retrieved for all identified reports of RCTs. Then, we constituted a sample of protocols reporting various pharmacologic interventions and non-pharmacologic treatments (surgery, implantable devices, rehabilitation, education, etc.; see sample size below).

### Experimental interventions

#### Objective of the tool

The writing aid tool based on CONSORT was developed to provide guidance to authors when writing a manuscript of a RCT evaluating pharmacologic treatment or non-pharmacologic treatment. The tool was individualized according to the type of treatment evaluated (drug, surgery, participative interventions such as rehabilitation, education).

#### Content of the tool

The content of the tool was based on the checklist and the Explanation and Elaboration document for CONSORT 2010 [3] and the checklists and the Explanation and Elaboration documents of the CONSORT extension for non-pharmacologic treatment [7]. For each domain, the tool comprised the corresponding CONSORT checklist item(s), bullet points with the key elements that need to be reported extracted from the Explanation and Elaboration document of the CONSORT 2010, and non-pharmacologic treatment extension, as well as (an) example(s) of good reporting. For the domain dedicated to the intervention, the bullet points and examples of adequate reporting were individualized according to the treatment evaluated (i.e. medication or treatment strategy; surgical procedures or devices; or participative interventions such as rehabilitation, education, behavioral treatment, or psychotherapy). For example, when the experimental treatment was a surgical procedure, the bullet points with the key elements that needed to be reported were specific to surgical procedures (e.g. anesthesia, preoperative care, postoperative care) and the examples of adequate reporting concerned surgical trials.

**Fig. 2:**
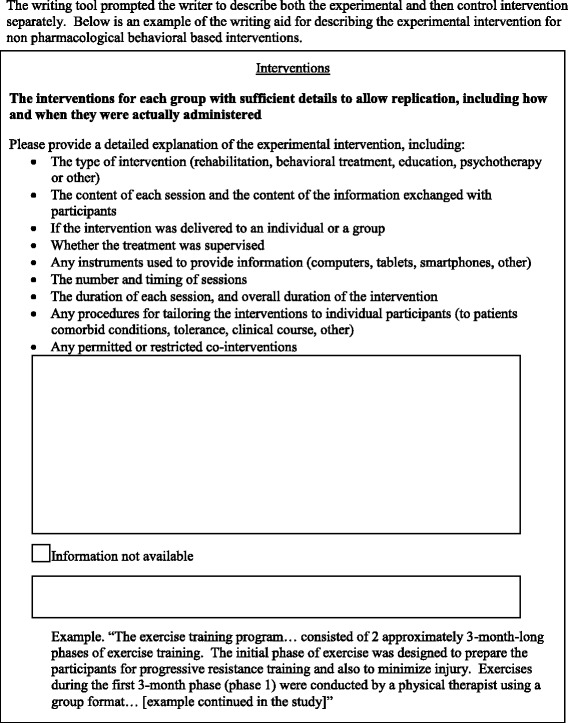
Example of the experimental writing tool

An example is included in Fig. 2. The entire tool is available at [28] (enter any username) and in Additional file 2.

#### Format of the tool

For each domain, the online writing aid tool consisted of a single or several large text boxes in which the participants could write the corresponding part of the methods section. Above each text box was a reminder of the information that should be reported. This reminder consisted of the related CONSORT item followed by the statement “Please describe” and bullet points with all the information that needed to be reported.

According to the domain and the complexity of the CONSORT item, the tool could contain one or several text boxes. For example, two boxes were dedicated to the domain trial design: one to describe the trial design and one to report important changes to methods after the trial commencements with reasons.

### Control intervention

For each domain, the intervention consisted of a large text box in which the participant could write this part of the methods section. They did not have the CONSORT checklist item.

### Writing session

Interventions were administered in the context of a practice writing session. Participants were asked to write six sections of a manuscript based on a protocol they were provided describing an RCT over a 4-hour period. Each participant was provided a protocol randomly selected in our sample of protocol in both electronic copy and paper copies. Two study monitors (CB and IB) supervised these writing sessions after providing a brief explanation of the task to be performed. Participants were aware that they would have access to the writing aid tool for some of the sections. They were not allowed to use any materials. Participants were told that all data would be anonymous and confidential.

For both the experimental and control interventions, participants were instructed to indicate any important or necessary information they would have wished to report that was not available in the provided study protocol (Fig. 2).

### Outcomes

#### Primary outcome

The primary outcome was the mean global score for completeness of reporting (scale 0–10) for all domains written with or without the writing aid tool.

For each domain, within each protocol selected, we pre-specified a series of keywords that should be reported. For example, in a study using a 1:1 randomization with a computer generated randomization list with blocks of four and stratification on the study site, the following key words were pre-specified ‘Computer generated’, ‘blocks of 4’, ‘1:1’, ‘stratification on site’. We also pre-specified a weight for each keyword.

For each protocol, completeness of reporting was determined by the presence or absence of the pre-specified keywords and their respective pre-specified weights. If the information was not available in the protocol but was described by the writer as missing, it was rated as completely reported.

Because the number of keywords varied among the domains by the domain type, the type of treatment evaluated, and the context of the protocol, we standardized the scores for each domain on a scale of 0–10. An example for the scoring system for completeness of reporting is in Additional file 3. Therefore, we obtained six scores for completeness of reporting for each participant-protocol pair, three associated with domains written with the writing tool and three with domains written without the tool. These scores were the unit of analysis (cf. Statistical methods section), and the statistical analysis allowed for estimating mean scores for completeness of reporting with and without the writing tool.

Two independent researchers blinded to intervention assignment and to the writer identity assessed the presence of these keywords for all protocols by domain. To maintain blinding, outcome assessors measured the outcome for each of the six domains separately, all text appearing in a different random order in the same format (with the same font and text size). After the researchers had assessed all the domains, they met to resolve any disagreements by consensus.

#### Secondary outcomes

Secondary outcomes were (1) the scores for completeness of reporting for each individual domain (trial design, randomization, blinding, participants, interventions, and outcomes of participant reports) and (2) the mean score for completeness of reporting of pre-specified essential elements of each domain (Additional file 4).

### Ancillary study

We aimed to compare the mean global score for completeness of reporting scores (scale 0–10) for all domains of the manuscript written by the participants with and without the tool to the methods sections of the published articles. For this purpose, we retrieved all the published reports corresponding to the selected protocols and their related appendices. The same two outcome assessors were asked to read the methods section of the published articles as well as all appendices referenced in the article and evaluate the presence or absence of the same pre-determined keywords. They were not blinded to the journal or authors’ names.

### Sample size calculation

The sample size was calculated using the same method as for a cluster randomized cross-over trial [29]. We assumed a mean score of 4 (0–10 scale) for the domains written without the tool (i.e. control group) and considered a standard deviation (SD) of 4 (which is a rather conservative assumption). Our hypothesis was that the mean score would be 6 for the domains written with the tool (i.e. experimental group). We specified an intraclass correlation coefficient of 0.8 (i.e. the correlation between scores of reports of two domains with the same interventional assignment, written by the same student). Such a conservative value was motivated by the nature of the design: two domains within a cluster are actually two domains completed by the same participant. We hypothesized that the intraclass correlation coefficient was half the intraclass correlation (0.4), and we considered a two-sided 5 % Type I error and a nominal power of 90 %. The inflation factor then was 1.4, the required number of observations (i.e. domains) per group 120, and the required number of participants 40.

### Statistical methods

Descriptive statistics were reported as number and percentage for categorical variables and median and interquartile range (IQR) for quantitative variables. The statistical unit of analysis was the section, which was embedded in the couple participant-protocol (since each participant had a different protocol, there is no distinction between participants and protocols). Therefore, we had six observations for each participant: three in the experimental group in which the writing aid tool was used, and three in the control group. For the main analysis, sections were considered exchangeable (i.e. the intervention effect was assumed to be the same whatever the domain of the section). Then, such a data-structure is the same as the classical data structure encountered in split-mouth designs or cluster randomized cross-over trials. Therefore, data were analyzed using a mixed model, which included a fixed intervention effect, a random participant effect, and a random participant-group effect [30].

Furthermore, because the hypothesis of a common intervention effect to all six domains was strong, we completed the primary analysis with a series of six independent substudies (i.e. one for each domain). For each of these substudies, we had only one statistical unit associated with each couple participant-protocol, which implies independence between the statistical units. Therefore, we performed classical Student *t*-tests. To evaluate the robustness of our results, we performed a sensitivity analysis with simulations of different possible weighting systems (Additional file 5).

For the ancillary study, we also considered the domain’s score for completeness of reporting as the unit of analysis. For each domain, within each protocol, we had one score for the participant of the present study and another for the authors of the published report. These paired data were split by whether the participant used the writing tool or not. Differences in paired scores were then analyzed in the framework of mixed models with no other fixed effect than an intercept and with the protocol as a random effect.

## Results

### Participants

**Fig. 3:**
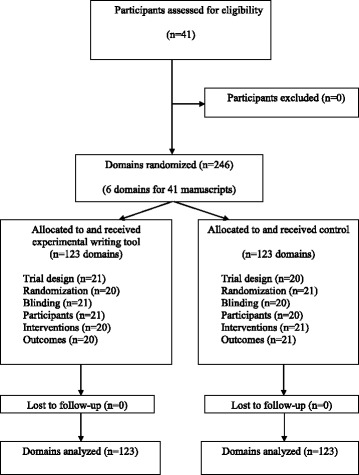
Flow diagram of participants and domain randomization

Forty-one masters and doctoral students participated in this study in May 2014. The flow diagram is shown in Fig. 3. As shown in Table 1, participants had a median age of 29 (IQR, 26–33) years. Seven participants (17 %) reported having had experience with writing about RCTs. Almost all participants (n = 38 (93 %)) reported having previously been taught about RCTs and 24 (58 %) reported being familiar with reporting guidelines.

**Table 1:** Participant and protocol characteristics

Participant characteristics	N = 41 (%)
Age, median (IQR)	29 (26–33)
Gender (female)	24 (58.5 %)
Education (doctoral students)	14 (34.1 %)
Frequency reading RCTs	
More than once a year	30 (73.2 %)
Once a month	6 (14.6 %)
Once a week	5 (12.2 %)
Experience writing RCTs	7 (17.1 %)
Taught about RCTs	38 (92.7 %)
Previously involved in RCTs	18 (43.9 %)
Familiar with guidelines	24 (58.5 %)
Comfortable with English	26 (63.4 %)
Trial characteristics	N = 41 (%)
Pharmacologic	24 (22.0 %)
Medication-based	19
Oral	7
Intravenous/parenteral	3
Intramuscular	2
Subcutaneous	3
Intradermal	1
Radiation	3
Strategy	4
Non-pharmacologic, surgical	8 (19.5 %)
Surgical procedures	4
Implantable devices	3
Non-pharmacologic, non-surgical	9 (58.5 %)
Care support	5
Psychotherapy	2
Patient education	2
External device	1
Physiotherapy	1

*RCT* Randomized controlled trial

### Protocols

We identified 158 of 308 citations screened, from which we sampled 41 protocols (one for each participant). A flowchart of the method for selecting protocols is shown in Additional file 1, and Table 1 describes the characteristics of protocols. Overall, 20 protocols reported an RCT evaluating pharmacologic treatments (oral drugs, intravenous/parenteral treatments, intramuscular treatments, subcutaneous treatments, intradermal, treatment strategies) and 17 non-pharmacologic treatments (surgery or implantable device, participative interventions, radiation).

### Outcomes

The mean global score for completeness of reporting (scale 0–10) for all domains was higher with than without the use of the writing aid tool: mean (SD) 7.1 (1.2) versus 5.0 (1.6), for a mean difference (95 % CI) of 2.1 (1.5–2.7; *P* <0.0001; Table 2).

**Table 2:** Completeness of reporting domains of methods sections with and without the writing aid tool

Domain	Writing aid tool scores (0–10)	No writing aid tool scores (0–10)	Mean difference (95 % CI)	*P* value
	Mean (SD)	Mean (SD)		
Global score for completeness of reporting for all domains (primary outcome)	7.1 (1.2)	5.0 (1.6)	2.1 (1.5–2.7)	<0.001*
Score for completeness of reporting for essential elements	7.8 (1.6)	6.4 (2.3)	1.4 (0.5–2.3)	0.002*
Completeness of reporting score by individual domain				
Trial design	8.1 (2.3)	2.7 (1.9)	5.4 (4.1–6.7)	<0.01**
Randomization	8.4 (2.4)	4.6 (2.9)	3.8 (1.1–4.4)	<0.01**
Blinding	6.9 (2.0)	6.2 (2.3)	0.7 (–0.7 to 2.0)	0.44**
Participants	6.7 (2.0)	4.5 (2.4)	2.2 (0.8–3.6)	<0.01**
Interventions	7.1 (1.5)	5.3 (2.0)	1.8 (0.7–2.9)	<0.01**
Outcomes	6.1 (2.1)	6.4 (3.0)	–0.3 (–2.0 to 1.3)	0.78**

*Mixed models

**Student *t*-test

Secondary outcomes are reported in Table 2. The completeness of reporting (score 0–10) was significantly higher with than without the writing aid tool for all domains except blinding and outcomes. The mean difference in scores (95 % CI) for each domain were for trial design, 5.4 (4.1–6.7), *P* <0.01; randomization, 3.8 (1.1–4.4), *P* <0.01; blinding, 0.7 (–0.7 to 2.0), *P* = 0.50; participants, 2.2 (0.8–3.6), *P* <0.01; interventions, 1.8 (0.7–2.9); *P* <0.01; and outcomes, –0.3 (–2.0 to 1.3), *P* = 0.43.

The completeness of reporting of essential elements was higher with than without the writing aid tool, with a mean difference (95 % CI) of 1.4 (0.5–2.3; *P* = 0.002).

On sensitivity analyses, the results were robust to the choice of the weight (Additional file 4).

### Ancillary study

Results of the ancillary study are shown in Table 3. Overall, the global completeness of reporting scores for the sections written without the writing aid tool did not significantly differ from those of the published manuscripts, with a mean difference of –0.48 (–1.07 to 0.11). In contrast, the global score for completeness of reporting scores for the sections written with the writing aid tool were higher than those of the publications, with a mean difference of +1.73 (1.10–2.37), *P* <0.001.

**Table 3:** Ancillary study, mean global score for completeness of reporting (scale 0–10) for all domains written with or without the writing aid tool compared to the published report

	Manuscript written by participants	Published report	Mean difference (95 % CI) in global score	*P* value^*^
	Mean (SD) global score	Mean (SD) global score	N = 123 domains	
	N = 123 domains	N = 123 domains		
No writing aid tool	5.02 (2.73)	5.50 (2.56)	–0.48 (–1.07 to 0.11)	0.11
Writing aid tool	7.10 (2.12)	5.36 (2.54)	1.73 (1.10–2.37)	<0.001

*Mixed models

## Discussion

We developed a writing aid tool to support the use of the CONSORT statement. We performed a proof-of-concept RCT including masters and doctoral students to evaluate the impact of this tool. Our results revealed a large effect of the writing tool on the completeness of reporting scores, with a mean difference in scores of 2.1 on a 0–10 scale. Completeness of reporting was significantly improved for all six domains of the methods section except for blinding and outcomes. However, for these domains, the scores without the use of the tool were relatively high and the opportunity for improvement was consequently limited. The ancillary study showed that the manuscripts written with the tool in a limited time (4 hours) had higher global score of completeness of reporting than the actual reports, which were published in high-impact-factor journals.

Our study has several strengths. First, it is a proof-of-concept study that used a strong design, an RCT, to evaluate the impact of this tool. We performed an RCT using a split-manuscript design. Second, this study explored the impact of the writing tool using real protocols of published RCTs. Third, the protocols selected evaluated a wide variety of different interventions exploring pharmacologic- and non-pharmacologic-based treatments (surgical and non-surgical) as well as various treatment strategies (treatment timing, target outcomes, etc.). The quality of reporting of the selected protocols varied.

However, our study had some limitations. First, the participants were masters and doctoral students and the writing session lasted only 4 hours, which is not representative of typical authors and the typical context of writing a report of an RCT. However, the global score for completeness of reporting was not lower than that for the published report. In contrast, this comparison confirmed a large effect of the writing tool. Second, the writing aid tool considered only six domains of the methods section of a manuscript using the CONSORT 2010 statement and the extension for non-pharmacologic treatments. We did not explore the impact of the implementation of all items of the CONSORT statement and all its extensions on the completeness of reporting. Third, there is an important degree of relatedness between the experimental intervention (the writing aid tool) and the evaluation. However, the primary outcome we used (i.e. the completeness of reporting according to the CONSORT statement and CONSORT extension for non-pharmacologic treatments) is the outcome usually used to evaluate the adherence to reporting guidelines and seemed the most relevant outcome. Finally, we lack a validated outcome measure to appropriately evaluate the quality of reporting in various contexts. However, the sensitivity analysis exploring the impact of a variation of the weight used showed that our results were robust.

Previous studies have explored the impact of other types of interventions on compliance with the CONSORT guidelines when reporting RCTs. These studies have explored the impact of the publication of the main CONSORT guidelines and the CONSORT extensions studying reporting before and after [10] and over time (using a time series analysis) [20], journal endorsement of CONSORT, and implementation by a journal (i.e. requiring writers to submit a completed checklist indicating where the information is reported in the manuscript) [18, 31]. Hopewell et al. [20] showed that an active policy to enforce the CONSORT guidelines for abstracts led to an immediate increase in the completeness of reporting. Finally, the results of the ancillary study need to be interpreted with caution. In fact, we did not evaluate the readability of each section. Some sections written with the tool could be more complete but also more difficult to read. Furthermore, outcome assessors were not blinded.

In this study, we proposed a new approach to improve the reporting of RCT results and assist with the successful implementation of the CONSORT statement. In fact, most of the initiatives previously proposed to improve implementation occur after the manuscript has been written. With this writing aid tool, we aimed (1) to guide writers to use the CONSORT guidelines and (2) to oblige writers to address each item of the CONSORT guidelines while writing the first draft of their manuscript. This study has important implications for authors, editors, and researchers. It demonstrates that a writing aid tool can improve the completeness of reporting and that we need to rethink the strategies used to implement CONSORT. The tool needs to be expanded to include all CONSORT items and incorporate all extensions. The tool is freely available on the web [28] and in Additional file 2.

## Conclusions

This study demonstrates that the use of a writing aid tool improves the completeness of reporting the results of RCTs. Our results indicate new opportunities to improve the quality of reporting research.

## Additional files

Additional file 1:
**Selection of protocols.** (DOCX 278 kb) Additional file 2:
**The CONSORT-based writing tool.** (DOCX 144 kb) Additional file 3:
**The CONSORT-based writing tool.** (DOCX 19 kb) Additional file 4:
**Weights for the scoring system for the completeness of reporting essential items according to intervention type.** (DOCX 18 kb) Additional file 5:
**Sensitivity analyses.** (DOCX 257 kb)

## Abbreviations

IQR: Interquartile range
RCTs: Randomized controlled trials
