# Supplementary material for: Impact of an online writing aid tool for writing a randomized trial report: the COBWEB (Consort-based WEB tool) randomized controlled trial
Source: BMC Med. 2015 Sep 15;13:221. doi: 10.1186/s12916-015-0460-y (PMC4570037; doi:10.1186/s12916-015-0460-y)

**Additional file 1**

*Selection of protocols*

We searched Pubmed for all RCTs published in NEJM and JCO between January 1, 2013 and March 28, 2014. We chose these journals because they provide access to the protocol for all the RCTs they publish. One researcher (CB) screened all titles and abstracts to select reports of two arm parallel group RCTs pertaining to either pharmacological or non-pharmacological treatments.

The final versions of protocols for all selected randomized controlled trials were retrieved and screened by this same researcher to select only those with a minimum amount of information (including at least one element of information requested by the writing tool for at least 5 of the 6 domains), and available in English. From all identified protocols, a subset of protocols were selected for study participants, including approximately half thought to be more conducive to the main CONSORT statement and half thought to be more conducive to the extension for nonpharmacologic treatments (around a quarter for surgical and a quarter for nonsurgical). Selected protocols were validated by two researchers experienced in methodology (IB and PR).

Figure 1. Flowchart for protocol selection


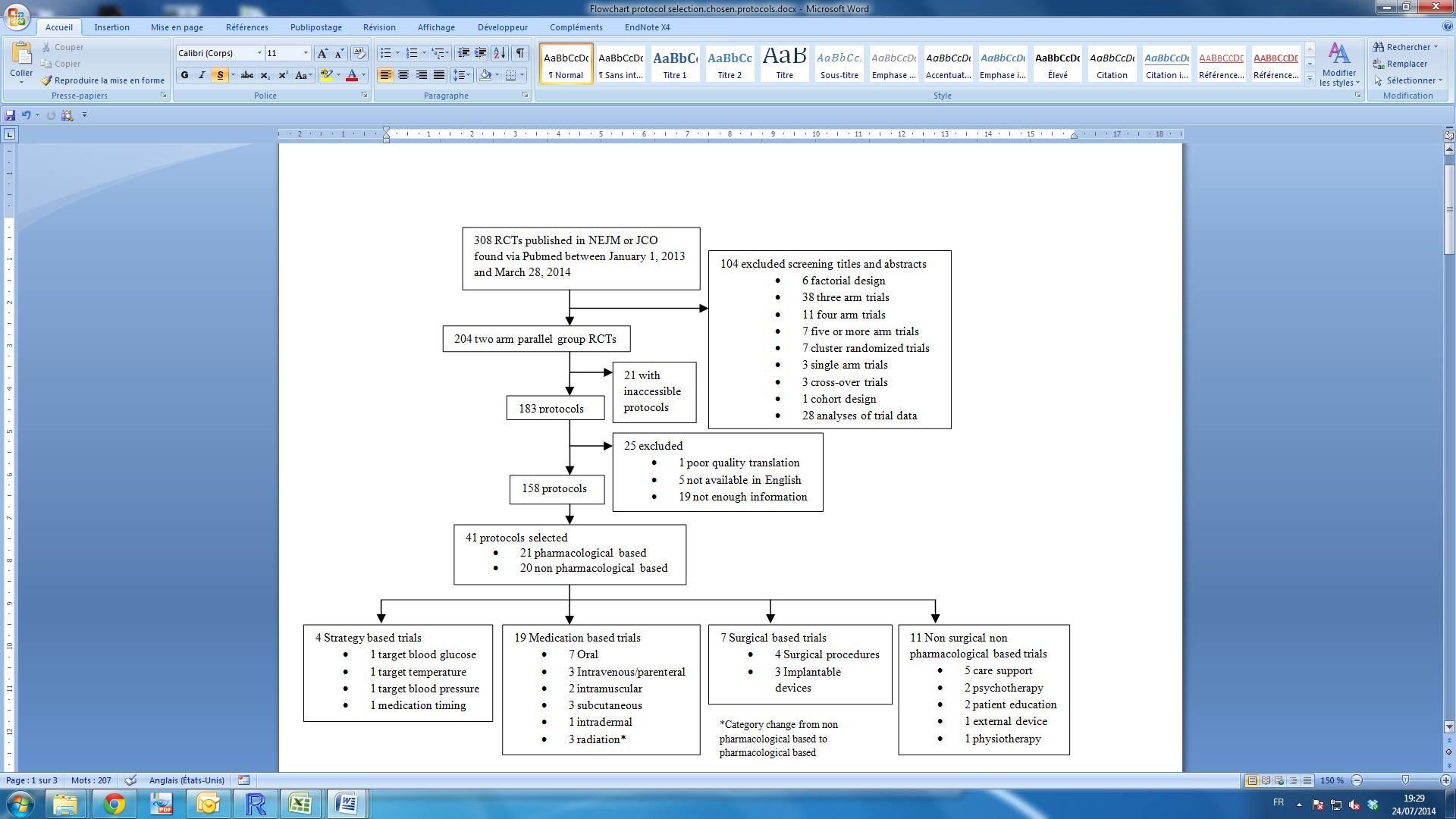

Supplement: Additional file 1: — Selection of protocols. (DOCX 278 kb) [file 12916_2015_460_MOESM1_ESM.docx]
