# Supplementary material for: Impact of an online writing aid tool for writing a randomized trial report: the COBWEB (Consort-based WEB tool) randomized controlled trial
Source: BMC Med. 2015 Sep 15;13:221. doi: 10.1186/s12916-015-0460-y (PMC4570037; doi:10.1186/s12916-015-0460-y)
Supplement: Additional file 2: — The CONSORT-based writing tool. (DOCX 144 kb) [file 12916_2015_460_MOESM2_ESM.docx]

**Additional file 2: The CONSORT based writing tool**

**Appendix 2A: The CONSORT based writing tool for pharmacological therapies**

**Trial design**

**Description of trial design (such as parallel, factorial) including allocation ratio**

Please describe:

- The type of trial design (parallel group, multi-arm, factorial, crossover, split-body, other)
- The conceptual framework (superiority, non-inferiority, equivalence, other)
- The allocation ratio
- Any other pertinent information (for drug development (phase 1,2,3), other)
- Information not available__________________________________

Example. “This was a parallel-group study with imbalanced randomisation [2:1]).”

**Important changes to methods after trial commencement (such as eligibility criteria), with reasons**

Please describe any changes made to:

- treatment regimens
- eligibility criteria
- allocation ratio
- duration of follow-up
- trial conduct (such as dropping a centre with poor data quality)
- any other important changes
- Not applicable
- Information not available__________________________________________

Example. **“**Patients were randomly assigned to one of six parallel groups, initially in 1:1:1:1:1:1 ratio, to receive either one of five otamixaban … regimens … or an active control of unfractionated heparin … an independent Data Monitoring Committee reviewed unblinded data for patient safety; no interim analyses for efficacy or futility were done. **During the trial, this committee recommended that the group receiving the lowest dose of otamixaban (0·035 mg/kg/h) be discontinued because of clinical evidence of inadequate anticoagulation. The protocol was immediately amended in accordance with that recommendation, and participants were subsequently randomly assigned in 2:2:2:2:1 ratio to the remaining otamixaban and control groups, respectively.”**

**Randomisation (PT)**

**Sequence generation, method used to generate the random allocation sequence**

- Please describe:
  - The method used to generate the allocation sequence
    - Methods used to generate the random allocation sequence (usually random number table or computerized random number generator)
    - Or another non random but generally accepted method (ie minimization)
  - The randomisation ratio (specify if randomization ratio is unequal 2:1, 3:1, other)
- Information not available_________________________________

**Example**. "Independent pharmacists dispensed either active or placebo inhalers according to a computer generated randomisation list."

**Sequence generation, type of randomisation, details of any restriction (such as blocking and block size)**

- If a restriction method was used, please:
  - Describe any permuted block randomisation if used (choice of block size, fixed or randomly varied, how they were generated)
  - Present any stratification variables (ie recruitment site, gender, years of experience, other…) with cut-off values when appropriate
  - Describe which variables were incorporated into any minimization scheme that may have been used, and indicate any random element(s)
- If no restriction method was used, please specify "simple randomisation"
- Information not available_________________________________

Example. “Randomization sequence was created using Stata 9.0 (StataCorp, College Station, TX) statistical software and was stratified by center with a 1:1 allocation using random block sizes of 2, 4, and 6."

**Allocation concealment, Mechanism used to implement the random allocation sequence (such as sequentially numbered containers), describing any steps taken to conceal the sequence until interventions were assigned**

- Please describe how the care provider enrolling patients was made ignorant of the next assignment in the sequence (different from blinding), such as through the:
  - Generation of the list “live” by an independent third party (use of a central telephone randomization system, automated assignment system, other)
  - Hiding of the treatment assignment sequence and prevention of tampering with this list by having a third party prepare and hide the allocation assignment in advance (for example via numbered identical bottles; or sequentially numbered, sealed, opaque envelopes)
- Information not available_____________________________________________

Example. “Treatments were centrally assigned on telephone verification of the correctness of inclusion criteria . . .”

“Treatments were placed in sequentially numbered containers. All of the containers were tamper-proof, equal in weight, and similar in appearance.”

**Implementation, who generated the random allocation sequence, who enrolled participants, and who assigned participants to interventions**

Please present:

- Who generated the random allocation sequence
- Who enrolled participants
- Who assigned interventions to the participants
- How the list of the assignment sequence was kept secret from those enrolling participants and assigning interventions
- Information not available_____________________________________________

Example. “Determination of whether a patient would be treated by streptomycin and bed-rest (S case) or by bed-rest alone (C case) was made by reference to a statistical series based on random sampling numbers drawn up for each sex at each centre by Professor Bradford Hill; the details of the series were unknown to any of the investigators or to the coordinator … After acceptance of a patient by the panel, and before admission to the streptomycin centre, the appropriate numbered envelope was opened at the central office; the card inside told if the patient was to be an S or a C case, and this information was then given to the medical officer of the centre."

“The various placebo and treatment blocks were then issued with a medication number and assigned to consecutive patients in a sequential order. Two copies of the randomisation list were prepared: one was used by the packaging department, . . . supplied in blister packs containing 20 capsules for morning and evening administration over 10 days. These blister packs were supplied in labeled boxes—ie, one box for each patient and each dose.”

**Blinding (PT)**

**If done, who was blinded after assignment to interventions (for example, participants, care providers, those assessing outcomes) and how**

- Please state who was blinded to treatment assignments and if so how
  - Study participant
  - Healthcare provider(s)
  - Data collectors
  - Outcome adjudicator(s)
  - Data analysts
  - Others
- Not applicable _______________________________________
- Information not available_________________________________

Examples. "Whereas patients and physicians allocated to the intervention group were aware of the allocated arm, outcome assessors and data analysts were kept blinded to the allocation."

**If relevant, description of the similarity of interventions**

Please describe or report:

- Any similarities or differences of the characteristics of the interventions (for pharmacological treatments appearance, taste, smell, method of administration, other)
- Any known compromises in blinding (ie was it necessary to unblind any participants at any point during the conduct of the trial?)
- Not applicable
- Information not available_________________________________

Example. “Jamieson Laboratories Inc provided 500-mg immediate release niacin in a white, oblong, bisect caplet. We independently confirmed caplet content using high performance liquid chromatography … The placebo was matched to the study drug for taste, color, and size, and contained microcrystalline cellulose, silicon dioxide, dicalcium phosphate, magnesium stearate, and stearic acid.”

**Participants (PT)**

**Eligibility criteria for participants**

Please describe the:

- Inclusion criteria (any age or gender restrictions, any necessary diagnostic criteria, other)
- Exclusion criteria (any clinical contraindications related to medical conditions or treatment contraindications, other)
- Methods of recruitment (referral or self selection, through advertisements, other)
- Information not available_________________________________

**Example**. "Eligible participants were all adults aged 18 or over with HIV who met the eligibility criteria for antiretroviral therapy according to the Malawian national HIV treatment guidelines (WHO clinical stage III or IV or any WHO stage with a CD4 count <250/mm3) and who were starting treatment with a BMI <18.5. Exclusion criteria were pregnancy and lactation or participation in another supplementary feeding programme."

**Settings and locations where data were collected**

Please indicate:

- If the study was a single or multicenter study (if multicenter, include the number of centers)
- The location(s) where the study was carried out (city, region, country…)
- Where patients were recruited (at a medical visit (general medicine doctor, specialist, other), at a hospital; from the community; other)
- Other important aspects of the setting (social, cultural, economic environment, climate, other)
- Any information about the settings and locations that could have influenced the observed results (problems with transportation that might have affected patient participation or delays in administering interventions)
- Information not available_________________________________________

Example. **“** The study took place at the antiretroviral therapy clinic of Queen Elizabeth Central Hospital in Blantyre, Malawi. Blantyre is the major commercial city of Malawi, with a population of 1 000 000 and an estimated HIV prevalence of 27% in adults in 2004."

**Interventions (PT)**

**The interventions for each group with sufficient details to allow replication, including how and when they were actually administered**

- Please provide a detailed explanation of the experimental intervention, including the:
  - Medication name (including main molecular component)
  - Mode of administration (oral, intravenous, subcutaneous, other)
  - Dose and duration of administration
  - Number and timing of medication administrations
  - Titration regimen if applicable (conditions for necessary treatment modifications and how these modifications were implemented)
  - Person administering the treatment (self administered, nurse, doctor, other…)
  - Any permitted or restricted co-interventions
- Information not available_________________________________

**Example**. "In POISE, patients received the first dose of the study drug (i.e., oral extended-release metoprolol 100 mg or matching placebo) 2-4 h before surgery. Study drug administration required a heart rate of 50 bpm or more and a systolic blood pressure of 100 mm Hg or greater; these haemodynamics were checked before each administration. If, at any time during the first 6 h after surgery, heart rate was 80 bpm or more and systolic blood pressure was 100 mm Hg or higher, patients received their first postoperative dose (extended-release metoprolol 100 mg or matched placebo) orally. If the study drug was not given during the first 6 h, patients received their first postoperative dose at 6 h after surgery. 12 h after the first postoperative dose, patients started taking oral extended-release metoprolol 200 mg or placebo every day for 30 days. If a patient’s heart rate was consistently below 45 bpm or their systolic blood pressure dropped below 100 mm Hg, study drug was withheld until their heart rate or systolic blood pressure recovered; the study drug was then restarted at 100 mg once daily. Patients whose heart rate was consistently 45-49 bpm and systolic blood pressure exceeded 100 mm Hg delayed taking the study drug for 12 h."

- Please provide a detailed explanation of the control intervention, including:
  - If the control was a placebo, otherwise indicate the control group medication name (including main molecular component)
  - If the control group received “usual care”, describe thoroughly what that constituted
  - If the control group or intervention group received a combination of interventions:
    - provide a thorough description of each intervention
    - present the order in which the combination of interventions were introduced or withdrawn, and if applicable the triggers for their introduction
  - Any differences in medication administration (mode, dose, timing, duration, titration, other)
  - Who administered the control treatment (if different from the experimental intervention)
- Information not available_____________________________________________

**Example**. "In POISE, patients received the first dose of the study drug (ie, oral extended-release metoprolol 100 mg **or matching placebo**) 2-4 h before surgery. Study drug administration required a heart rate of 50 bpm or more and a systolic blood pressure of 100 mm Hg or greater; these haemodynamics were checked before each administration. If, at any time during the first 6 h after surgery, heart rate was 80 bpm or more and systolic blood pressure was 100 mm Hg or higher, patients received their first postoperative dose (extended-release metoprolol 100 mg **or matched placebo**) orally. If the study drug was not given during the first 6 h, patients received their first postoperative dose at 6 h after surgery. 12 h after the first postoperative dose, patients started taking oral extended-release metoprolol 200 mg or placebo every day for 30 days. If a patient’s heart rate was consistently below 45 bpm or their systolic blood pressure dropped below 100 mm Hg, study drug was withheld until their heart rate or systolic blood pressure recovered; the study drug was then restarted at 100 mg once daily. Patients whose heart rate was consistently 45-49 bpm and systolic blood pressure exceeded 100 mm Hg delayed taking the study drug for 12 h."

**Outcomes**

**Completely defined pre-specified primary and secondary outcome measures, including how and when they were assessed**

Please provide a detailed description of the primary outcome(s), by describing:

- - The variable of interest (ie pain, quality of life, clinical improvement, clinical event, other)
  - How the variable was defined (ie proportion of pain scores over 7, average quality of life score, change in blood pressure, number of myocardial infarctions, other)
  - How the variable was measured (ie visual analog scale, SF-36, systolic blood pressure, according to the WHO diagnostic criteria, other)
  - The overall time frame indicating pre-specified time point(s) (difference from baseline to 3 months; average over 1 year (at baseline, 6, and 12 months); final values at 1 month; time to event within 30 days; time to event or pre-specified final day of follow-up [indicate date]; other)
  - Who assessed the outcome (the patient, doctor, nurse, caretaker, other)
  - If any special skills or training were required for the outcome assessment
- Information not available_________________________________

**Example 1**. "The primary endpoint with respect to efficacy in psoriasis was the proportion of patients achieving a 75% improvement in psoriasis activity from baseline to 12 weeks as measured by the PASI [psoriasis area and severity index].”

Please provide a detailed description of the secondary outcome(s), by describing:

- - The variable of interest (ie pain, quality of life, clinical improvement, clinical event, other)
  - How the variable was defined (ie proportion of pain scores over 7, average quality of life score, change in blood pressure, number of myocardial infarctions, other)
  - How the variable was measured (ie visual analog scale, SF-36, systolic blood pressure, according to the WHO diagnostic criteria, other)
  - The overall time frame indicating pre-specified time point(s) (difference from baseline to 3 months; average over 1 year (at baseline, 6, and 12 months); final values at 1 month; time to event within 30 days; time to event or pre-specified final day of follow-up [indicate date]; other)
  - Who assessed the outcome (the patient, doctor, nurse, caretaker, other)
  - If any special skills or training were required for the outcome assessment
- Information not available_________________________________

**Example 2**. “Participants underwent a series of assessments at baseline and at 3 and 6 months after baseline using standardized procedures and forms. Follow-up data collection at 3 and 6 months was attempted for all participants, even those who had discontinued the therapy to which they had been assigned or who had received less than the full dose of exercise. Secondary outcomes: Participants also underwent assessments of muscle strength, gait, balance, body composition, and quality of life. Maximal voluntary muscle strength for knee extension and flexion of the fractured and unfractured limbs was measured using Cybex isokinetic dynamometry (Cybex International, Medway, Mass). Gait speed was measured for the middle 7 m of a 12-m walkway at the participant's self-selected and maximal gait speed. Balance was measured with the Progressive Romberg Test, Berg Balance Instrument, and a timed single-limb stance. Total body dual-energy x-ray absorptiometry (DEXA) (Hologic QDR1000/W, software version 6.2OD, Waltham, Mass) was used to assess total and regional fat-free mass and bone mineral density (BMD). The Medical Outcomes Study Short-Form 36 (SF-36) instrument and a modified version of the Hip Rating Questionnaire were administered to measure quality of life. The procedures for these measurements have been described in detail.  Except for DEXA measurement, the research staff who conducted the assessments were not involved in exercise training and were blinded to group assignment.”

**Any changes to trial outcomes after the trial commenced, with reasons**

Please describe any:

- Unplanned changes to: eligibility criteria, interventions, examinations, data collection, methods of analysis, and outcomes.
- Justification for unplanned changes (external evidence from other trials or systematic reviews suggesting the end point might not be appropriate, or recruitment or the overall event rate in the trial may be lower than expected, other)
- Not applicable
- Information not available_____________________________________________

Example. "The original primary endpoint was all-cause mortality, but, during a masked analysis, the data and safety monitoring board noted that overall mortality was lower than had been predicted and that the study could not be completed with the sample size and power originally planned. **The steering committee therefore decided to adopt co-primary endpoints of all-cause mortality (the original primary endpoint), together with all-cause mortality or cardiovascular hospital admissions (the first prespecified secondary endpoint)**."

**Appendix 2B: The CONSORT based writing tool for nonpharmacological therapies, mostly surgical oriented**

**Trial design**

**Description of trial design (such as parallel, factorial) including allocation ratio**

Please describe:

- The type of trial design (parallel group, multi-arm, factorial, crossover, split-body, other)
- The conceptual framework (superiority, non-inferiority, equivalence, other)
- The allocation ratio
- Any other pertinent information (for drug development (phase 1,2,3), other)
- Information not available__________________________________

Example. “This was a parallel-group study with imbalanced randomisation [2:1]).”

**Important changes to methods after trial commencement (such as eligibility criteria), with reasons**

Please describe any changes made to:

- treatment regimens
- eligibility criteria
- allocation ratio
- duration of follow-up
- trial conduct (such as dropping a centre with poor data quality)
- any other important changes
- Not applicable
- Information not available__________________________________________

Example. **“**Patients were randomly assigned to one of six parallel groups, initially in 1:1:1:1:1:1 ratio, to receive either one of five otamixaban … regimens … or an active control of unfractionated heparin … an independent Data Monitoring Committee reviewed unblinded data for patient safety; no interim analyses for efficacy or futility were done. **During the trial, this committee recommended that the group receiving the lowest dose of otamixaban (0·035 mg/kg/h) be discontinued because of clinical evidence of inadequate anticoagulation. The protocol was immediately amended in accordance with that recommendation, and participants were subsequently randomly assigned in 2:2:2:2:1 ratio to the remaining otamixaban and control groups, respectively.”**

**Randomisation (NPT)**

**Sequence generation, method used to generate the random allocation sequence**

- Please describe:
  - The method used to generate the allocation sequence
    - Methods used to generate the random allocation sequence (usually random number table or computerized random number generator)
    - Or another non random but generally accepted method (ie minimization)
  - The randomisation ratio (specify if randomization ratio is unequal 2:1, 3:1, other)
- Information not available_________________________________

**Example**. "Independent pharmacists dispensed either active or placebo inhalers according to a computer generated randomisation list."

**Sequence generation, type of randomisation, details of any restriction (such as blocking and block size)**

- If a restriction method was used, please:
  - Describe any permuted block randomisation if used (choice of block size, fixed or randomly varied, how they were generated)
  - Present any stratification variables (ie recruitment site, gender, years of experience, other…) with cut-off values when appropriate
  - Describe which variables were incorporated into any minimization scheme that may have been used, and indicate any random element(s)
- If no restriction method was used, please specify "simple randomisation"
- Information not available_________________________________

Example. “Randomization sequence was created using Stata 9.0 (StataCorp, College Station, TX) statistical software and was stratified by center with a 1:1 allocation using random block sizes of 2, 4, and 6."

**Allocation concealment, Mechanism used to implement the random allocation sequence (such as sequentially numbered containers), describing any steps taken to conceal the sequence until interventions were assigned**

- Please describe how the care provider enrolling patients was made ignorant of the next assignment in the sequence (different from blinding), such as through the:
  - Generation of the list “live” by an independent third party (use of a central telephone randomization system, automated assignment system, other)
  - Hiding of the treatment assignment sequence and prevention of tampering with this list by having a third party prepare and hide the allocation assignment in advance (for example via numbered identical bottles; or sequentially numbered, sealed, opaque envelopes)
- Information not available_____________________________________________

Example. “Treatments were centrally assigned on telephone verification of the correctness of inclusion criteria . . .”

“Treatments were placed in sequentially numbered containers. All of the containers were tamper-proof, equal in weight, and similar in appearance.”

**Implementation, who generated the random allocation sequence, who enrolled participants, and who assigned participants to interventions**

Please present:

- Who generated the random allocation sequence
- Who enrolled participants
- Who assigned interventions to the participants
- How the list of the assignment sequence was kept secret from those enrolling participants and assigning interventions
- Information not available_____________________________________________

Example. “Determination of whether a patient would be treated by streptomycin and bed-rest (S case) or by bed-rest alone (C case) was made by reference to a statistical series based on random sampling numbers drawn up for each sex at each centre by Professor Bradford Hill; the details of the series were unknown to any of the investigators or to the coordinator … After acceptance of a patient by the panel, and before admission to the streptomycin centre, the appropriate numbered envelope was opened at the central office; the card inside told if the patient was to be an S or a C case, and this information was then given to the medical officer of the centre."

“The various placebo and treatment blocks were then issued with a medication number and assigned to consecutive patients in a sequential order. Two copies of the randomisation list were prepared: one was used by the packaging department, . . . supplied in blister packs containing 20 capsules for morning and evening administration over 10 days. These blister packs were supplied in labeled boxes—ie, one box for each patient and each dose.”

**How care providers were allocated to each trial group**

Please present how healthcare providers were allocated to trial groups:

- By randomization
- According to expertise or preference (expertise-based RCT)
- By any other means
- Not applicable
- Information not available_____________________________________________

Examples. “At each of the 12 sites, 4 female therapists were randomly assigned to deliver either PE [prolonged exposure]or PCT [present-centered therapy] (n=2 per condition per site)… By design, each therapist treats 10 participants: 2 training cases during a 6-month run-up period, and 8 randomized cases during 2 years of recruitment.”

“The patients were randomly selected for one of two operative procedures: open reduction and internal fixation or external fixation and limited internal fixation. The six attending orthopaedic surgeons who performed the operations had been assigned to a treatment group according to their expertise or their preference with regard to fixation. Each patient was managed by the one of the six surgeons who was on call when the patient was seen in the emergency room.”

**Blinding (NPT)**

**If done, who was blinded after assignment to interventions (for example, participants, care providers, those assessing outcomes) and how**

- Please state who was blinded to treatment assignments and if so how
  - Study participant
  - Healthcare provider(s)
  - Those administering co-interventions
  - Data collectors
  - Outcome adjudicator(s)
  - Data analysts
  - Others
- Not applicable _______________________________________
- Information not available_________________________________

Examples. "Whereas patients and physicians allocated to the intervention group were aware of the allocated arm, outcome assessors and data analysts were kept blinded to the allocation."

**If relevant, description of the similarity of interventions**

Please describe or report:

- Any similarities or differences of the characteristics of the interventions (for pharmacological treatments appearance, taste, smell, method of administration, other)
- Any known compromises in blinding (ie was it necessary to unblind any participants at any point during the conduct of the trial?)
- Not applicable
- Information not available_________________________________

Example. “Jamieson Laboratories Inc provided 500-mg immediate release niacin in a white, oblong, bisect caplet. We independently confirmed caplet content using high performance liquid chromatography … The placebo was matched to the study drug for taste, color, and size, and contained microcrystalline cellulose, silicon dioxide, dicalcium phosphate, magnesium stearate, and stearic acid.”

**Participants (NPT)**

**Eligibility criteria for participants**

Please describe the:

- Inclusion criteria (any age or gender restrictions, any necessary diagnostic criteria, other)
- Exclusion criteria (any clinical contraindications related to medical conditions or treatment contraindications, other)
- Methods of recruitment (referral or self selection, through advertisements, other)
- Information not available_________________________________

**Example**. "Eligible participants were all adults aged 18 or over with HIV who met the eligibility criteria for antiretroviral therapy according to the Malawian national HIV treatment guidelines (WHO clinical stage III or IV or any WHO stage with a CD4 count <250/mm3) and who were starting treatment with a BMI <18.5. Exclusion criteria were pregnancy and lactation or participation in another supplementary feeding programme."

**Settings and locations where data were collected**

Please indicate:

- If the study was a single or multicenter study (if multicenter, include the number of centers)
- The location(s) where the study was carried out (city, region, country…)
- Where patients were recruited (at a medical visit (general medicine doctor, specialist, other), at a hospital; from the community; other)
- Other important aspects of the setting (social, cultural, economic environment, climate, other)
- Any information about the settings and locations that could have influenced the observed results (problems with transportation that might have affected patient participation or delays in administering interventions)
- Information not available_________________________________________

Example. **“** The study took place at the antiretroviral therapy clinic of Queen Elizabeth Central Hospital in Blantyre, Malawi. Blantyre is the major commercial city of Malawi, with a population of 1 000 000 and an estimated HIV prevalence of 27% in adults in 2004."

**Eligibility criteria for centers and those performing the interventions.**

Please describe the eligibility criteria:

- For centers:
  - center volume for the procedure of interest
  - other
- For care providers:
  - professional qualifications
  - years in practice
  - number of interventions performed
  - skill as assessed by level of complication
  - specific training before trial initiation
  - other
- Information not available_________________________________________

Example. “All participating centres… were major neurosurgical centres, treating large numbers of patients after aneurismal subarachnoid hemorrhage (SAH), each centre treating between 60 and 200 cases annually… Centres had to have expertise in both neurosurgical and endovascular management of ruptured aneurysms. Only accredited neurosurgeons with experience of aneurysm surgery were permitted to manage patients in the trial. Endovascular operators had to have done a minimum of 30 aneurysm treatment procedures, before they were permitted to treat patients in the trial.”

**Interventions (NPT surgery etc)**

**The interventions for each group with sufficient details to allow replication, including how and when they were actually administered**

Please provide a detailed explanation of the experimental intervention, including:

- - The type of the intervention (surgery, technical procedures or implantable devices)
  - Anesthesia management
  - Preoperative care
  - Intra-operative care including all different components of the treatment procedure
  - Postoperative care
  - Any devices or equipment used along with the configuration details for any device
  - Any co-interventions permitted or restricted
- Information not available_________________________________

Example. “Tidal irrigation (TI) and sham irrigation (SI) procedures began with cleansing of the skin with betadine. Topical anesthesia was produced with ethyl chloride spray, following which subcutaneous and capsular anesthesia was induced with 3-5 ml 1% lidocaine, injected at the medial midpatellar and lateral suprapatellar ports. The capsule was punctured, aspiration of up to 5 ml of synovial fluid was attempted, and intraarticular injection with 20 ml 0.25% bupivacaine was performed via the medical midpatellar port. A vertical drape was then raised to obscure the subject’s view of the remainder of the procedure. However, a 1-liter bag of sterile normal saline was hung at the edge of the drape within view of the subject. The knee was then draped with sterile towels and the connecting tubing and 3-way stop-cocks were assembled and attached to an empty 1-liter waster bag and a 50-ml syringe, producing a closed system for fluid delivery, aspiration, and ejection.

TI treatment. To administer SI, the 14-gauge needle was used to puncture the capsule at the lateral suprapatellar port, and the bupivacaine-containing fluid was aspirated and ejected to the waste bag. Fresh saline, in aliquots of 30-50 ml, was then injected in to the knee, withdrawn, and ejected repeatedly. The volume of each aliquot was 50 ml unless limited by the capacity of the knee, as assessed by back-pressure, capsular distension, or, occasionally, subject discomfort. The knee was maintained in relaxed extension throughout the procedure, and the anterior knee was massaged, as necessary, to facilitate the aspiration of the injected saline. After passage of 1 liter of saline through the knee, the joint was evacuated and the needle was removed.”

Please provide a detailed explanation of the control intervention, including:

- - The type of control intervention (placebo, usual care, an active treatment, a waiting list, other)
  - If the control group is to receive “usual care”, describe thoroughly what that constitutes
  - If the control group or intervention group is to receive a combination of interventions:
    - provide a thorough description of each intervention,
    - present the order in which the combination of interventions were introduced or withdrawn, and if applicable the triggers for their introduction
  - Any other differences for the control intervention (details of pre, intra and postoperative care, devices or equipment used, other)
  - If different from experimental intervention, any permitted or restricted co-interventions
- Information not available_____________________________________________

**Example**. "To administer SI [sham intervention], the 14-gauge needle was advanced to, but not through, the joint capsule via the lateral suprapatellar port. Fresh saline was drawn from the supply bag in aliquots of 40-50 ml, and 3-5 ml of saline was slowly clysed into the subcutaneous tissues with each mimicked “exchange” before the remainder of the saline was expelled into the waste gag. Positioning of the knee and anterior manipulation were performed as for actual TI [tidal irrigation]. After passage of 1 liter of saline through the tubing (and clysis of 50-1000 ml of saline into the subcutaneous tissue), the needle was removed.”

**Details of how the interventions were standardized.**

Please describe:

- Any standardization methods (training of care providers, equipment requirements, written manuals, specific guidelines, and materials used to train care providers to uniformly deliver the intervention)
- Where the reader may access materials used to standardize the intervention (by including a Web appendix with their article or a link to a stable Web site)
- Not applicable
- Information not available_____________________________________________

Example. “The usual practices of surgeons performing optic nerve decompression surgery were determined through literature review and through a survey of study surgeons. These practices were described in the protocol as a series of 31 steps, only six of which were required to be performed so as to ensure adequacy of the surgery as well as safety of the patient. The remaining steps could be performed according to surgeon preference as they did not directly affect either patient safety or adequacy of surgery. Each study surgeon signed a written commitment to adhere to the six required steps, which were: general anesthesia, medial approach, no mechanical static traction, subarachnoid dissection if no cerebrospinal fluid release was seen following fenestration of the optic nerve sheath, no more than 7 minutes of sustained traction on the globe at any one time and rest periods of at least 2 minutes following any 7-minute period of globe traction.”

**Details of how adherence of care providers with the protocol was assessed or enhanced.**

Please describe:

- Any means to assess or enhance care provider adherence (review of case report forms, videotapes, audiotapes, other)
- Not applicable
- Information not available___________________________________________

Example. “Qualitative analysis of the videos was carried out by one experienced SILS surgeon and one surgical trainee, blinded to the group and case number, marking the performance in line with objective structured assessment of technical skills (OSATS) based generic skills, the modified global rating scale (mGRS) was used. For procedure-specific rating scales (PSRS).

**Outcomes**

**Completely defined pre-specified primary and secondary outcome measures, including how and when they were assessed**

Please provide a detailed description of the primary outcome(s), by describing:

- - The variable of interest (ie pain, quality of life, clinical improvement, clinical event, other)
  - How the variable was defined (ie proportion of pain scores over 7, average quality of life score, change in blood pressure, number of myocardial infarctions, other)
  - How the variable was measured (ie visual analog scale, SF-36, systolic blood pressure, according to the WHO diagnostic criteria, other)
  - The overall time frame indicating pre-specified time point(s) (difference from baseline to 3 months; average over 1 year (at baseline, 6, and 12 months); final values at 1 month; time to event within 30 days; time to event or pre-specified final day of follow-up [indicate date]; other)
  - Who assessed the outcome (the patient, doctor, nurse, caretaker, other)
  - If any special skills or training were required for the outcome assessment
- Information not available_________________________________

**Example 1**. "The primary endpoint with respect to efficacy in psoriasis was the proportion of patients achieving a 75% improvement in psoriasis activity from baseline to 12 weeks as measured by the PASI [psoriasis area and severity index].”

Please provide a detailed description of the secondary outcome(s), by describing:

- - The variable of interest (ie pain, quality of life, clinical improvement, clinical event, other)
  - How the variable was defined (ie proportion of pain scores over 7, average quality of life score, change in blood pressure, number of myocardial infarctions, other)
  - How the variable was measured (ie visual analog scale, SF-36, systolic blood pressure, according to the WHO diagnostic criteria, other)
  - The overall time frame indicating pre-specified time point(s) (difference from baseline to 3 months; average over 1 year (at baseline, 6, and 12 months); final values at 1 month; time to event within 30 days; time to event or pre-specified final day of follow-up [indicate date]; other)
  - Who assessed the outcome (the patient, doctor, nurse, caretaker, other)
  - If any special skills or training were required for the outcome assessment
- Information not available_________________________________

**Example 2**. “Participants underwent a series of assessments at baseline and at 3 and 6 months after baseline using standardized procedures and forms. Follow-up data collection at 3 and 6 months was attempted for all participants, even those who had discontinued the therapy to which they had been assigned or who had received less than the full dose of exercise. Secondary outcomes: Participants also underwent assessments of muscle strength, gait, balance, body composition, and quality of life. Maximal voluntary muscle strength for knee extension and flexion of the fractured and unfractured limbs was measured using Cybex isokinetic dynamometry (Cybex International, Medway, Mass). Gait speed was measured for the middle 7 m of a 12-m walkway at the participant's self-selected and maximal gait speed. Balance was measured with the Progressive Romberg Test, Berg Balance Instrument, and a timed single-limb stance. Total body dual-energy x-ray absorptiometry (DEXA) (Hologic QDR1000/W, software version 6.2OD, Waltham, Mass) was used to assess total and regional fat-free mass and bone mineral density (BMD). The Medical Outcomes Study Short-Form 36 (SF-36) instrument and a modified version of the Hip Rating Questionnaire were administered to measure quality of life. The procedures for these measurements have been described in detail.  Except for DEXA measurement, the research staff who conducted the assessments were not involved in exercise training and were blinded to group assignment.”

**Any changes to trial outcomes after the trial commenced, with reasons**

Please describe any:

- Unplanned changes to: eligibility criteria, interventions, examinations, data collection, methods of analysis, and outcomes.
- Justification for unplanned changes (external evidence from other trials or systematic reviews suggesting the end point might not be appropriate, or recruitment or the overall event rate in the trial may be lower than expected, other)
- Not applicable
- Information not available_____________________________________________

Example. "The original primary endpoint was all-cause mortality, but, during a masked analysis, the data and safety monitoring board noted that overall mortality was lower than had been predicted and that the study could not be completed with the sample size and power originally planned. **The steering committee therefore decided to adopt co-primary endpoints of all-cause mortality (the original primary endpoint), together with all-cause mortality or cardiovascular hospital admissions (the first prespecified secondary endpoint)**."

**Appendix 2C: The CONSORT based writing tool for nonpharmacological therapies, mostly rehabilitation oriented**

**Trial design**

**Description of trial design (such as parallel, factorial) including allocation ratio**

Please describe:

- The type of trial design (parallel group, multi-arm, factorial, crossover, split-body, other)
- The conceptual framework (superiority, non-inferiority, equivalence, other)
- The allocation ratio
- Any other pertinent information (for drug development (phase 1,2,3), other)
- Information not available__________________________________

Example. “This was a parallel-group study with imbalanced randomisation [2:1]).”

**Important changes to methods after trial commencement (such as eligibility criteria), with reasons**

Please describe any changes made to:

- treatment regimens
- eligibility criteria
- allocation ratio
- duration of follow-up
- trial conduct (such as dropping a centre with poor data quality)
- any other important changes
- Not applicable
- Information not available__________________________________________

Example. **“**Patients were randomly assigned to one of six parallel groups, initially in 1:1:1:1:1:1 ratio, to receive either one of five otamixaban … regimens … or an active control of unfractionated heparin … an independent Data Monitoring Committee reviewed unblinded data for patient safety; no interim analyses for efficacy or futility were done. **During the trial, this committee recommended that the group receiving the lowest dose of otamixaban (0·035 mg/kg/h) be discontinued because of clinical evidence of inadequate anticoagulation. The protocol was immediately amended in accordance with that recommendation, and participants were subsequently randomly assigned in 2:2:2:2:1 ratio to the remaining otamixaban and control groups, respectively.”**

**Randomisation (NPT)**

**Sequence generation, method used to generate the random allocation sequence**

- Please describe:
  - The method used to generate the allocation sequence
    - Methods used to generate the random allocation sequence (usually random number table or computerized random number generator)
    - Or another non random but generally accepted method (ie minimization)
  - The randomisation ratio (specify if randomization ratio is unequal 2:1, 3:1, other)
- Information not available_________________________________

**Example**. "Independent pharmacists dispensed either active or placebo inhalers according to a computer generated randomisation list."

**Sequence generation, type of randomisation, details of any restriction (such as blocking and block size)**

- If a restriction method was used, please:
  - Describe any permuted block randomisation if used (choice of block size, fixed or randomly varied, how they were generated)
  - Present any stratification variables (ie recruitment site, gender, years of experience, other…) with cut-off values when appropriate
  - Describe which variables were incorporated into any minimization scheme that may have been used, and indicate any random element(s)
- If no restriction method was used, please specify "simple randomisation"
- Information not available_________________________________

Example. “Randomization sequence was created using Stata 9.0 (StataCorp, College Station, TX) statistical software and was stratified by center with a 1:1 allocation using random block sizes of 2, 4, and 6."

**Allocation concealment, Mechanism used to implement the random allocation sequence (such as sequentially numbered containers), describing any steps taken to conceal the sequence until interventions were assigned**

- Please describe how the care provider enrolling patients was made ignorant of the next assignment in the sequence (different from blinding), such as through the:
  - Generation of the list “live” by an independent third party (use of a central telephone randomization system, automated assignment system, other)
  - Hiding of the treatment assignment sequence and prevention of tampering with this list by having a third party prepare and hide the allocation assignment in advance (for example via numbered identical bottles; or sequentially numbered, sealed, opaque envelopes)
- Information not available_____________________________________________

Example. “Treatments were centrally assigned on telephone verification of the correctness of inclusion criteria . . .”

“Treatments were placed in sequentially numbered containers. All of the containers were tamper-proof, equal in weight, and similar in appearance.”

**Implementation, who generated the random allocation sequence, who enrolled participants, and who assigned participants to interventions**

Please present:

- Who generated the random allocation sequence
- Who enrolled participants
- Who assigned interventions to the participants
- How the list of the assignment sequence was kept secret from those enrolling participants and assigning interventions
- Information not available_____________________________________________

Example. “Determination of whether a patient would be treated by streptomycin and bed-rest (S case) or by bed-rest alone (C case) was made by reference to a statistical series based on random sampling numbers drawn up for each sex at each centre by Professor Bradford Hill; the details of the series were unknown to any of the investigators or to the coordinator … After acceptance of a patient by the panel, and before admission to the streptomycin centre, the appropriate numbered envelope was opened at the central office; the card inside told if the patient was to be an S or a C case, and this information was then given to the medical officer of the centre."

“The various placebo and treatment blocks were then issued with a medication number and assigned to consecutive patients in a sequential order. Two copies of the randomisation list were prepared: one was used by the packaging department, . . . supplied in blister packs containing 20 capsules for morning and evening administration over 10 days. These blister packs were supplied in labeled boxes—ie, one box for each patient and each dose.”

**How care providers were allocated to each trial group**

Please present how healthcare providers were allocated to trial groups:

- By randomization
- According to expertise or preference (expertise-based RCT)
- By any other means
- Not applicable
- Information not available_____________________________________________

Examples. “At each of the 12 sites, 4 female therapists were randomly assigned to deliver either PE [prolonged exposure]or PCT [present-centered therapy] (n=2 per condition per site)… By design, each therapist treats 10 participants: 2 training cases during a 6-month run-up period, and 8 randomized cases during 2 years of recruitment.”

“The patients were randomly selected for one of two operative procedures: open reduction and internal fixation or external fixation and limited internal fixation. The six attending orthopaedic surgeons who performed the operations had been assigned to a treatment group according to their expertise or their preference with regard to fixation. Each patient was managed by the one of the six surgeons who was on call when the patient was seen in the emergency room.”

**Blinding (NPT)**

**If done, who was blinded after assignment to interventions (for example, participants, care providers, those assessing outcomes) and how**

- Please state who was blinded to treatment assignments and if so how
  - Study participant
  - Healthcare provider(s)
  - Those administering co-interventions
  - Data collectors
  - Outcome adjudicator(s)
  - Data analysts
  - Others
- Not applicable _______________________________________
- Information not available_________________________________

Examples. "Whereas patients and physicians allocated to the intervention group were aware of the allocated arm, outcome assessors and data analysts were kept blinded to the allocation."

**If relevant, description of the similarity of interventions**

Please describe or report:

- Any similarities or differences of the characteristics of the interventions (for pharmacological treatments appearance, taste, smell, method of administration, other)
- Any known compromises in blinding (ie was it necessary to unblind any participants at any point during the conduct of the trial?)
- Not applicable
- Information not available_________________________________

Example. “Jamieson Laboratories Inc provided 500-mg immediate release niacin in a white, oblong, bisect caplet. We independently confirmed caplet content using high performance liquid chromatography … The placebo was matched to the study drug for taste, color, and size, and contained microcrystalline cellulose, silicon dioxide, dicalcium phosphate, magnesium stearate, and stearic acid.”

**Participants (NPT)**

**Eligibility criteria for participants**

Please describe the:

- Inclusion criteria (any age or gender restrictions, any necessary diagnostic criteria, other)
- Exclusion criteria (any clinical contraindications related to medical conditions or treatment contraindications, other)
- Methods of recruitment (referral or self selection, through advertisements, other)
- Information not available_________________________________

**Example**. "Eligible participants were all adults aged 18 or over with HIV who met the eligibility criteria for antiretroviral therapy according to the Malawian national HIV treatment guidelines (WHO clinical stage III or IV or any WHO stage with a CD4 count <250/mm3) and who were starting treatment with a BMI <18.5. Exclusion criteria were pregnancy and lactation or participation in another supplementary feeding programme."

**Settings and locations where data were collected**

Please indicate:

- If the study was a single or multicenter study (if multicenter, include the number of centers)
- The location(s) where the study was carried out (city, region, country…)
- Where patients were recruited (at a medical visit (general medicine doctor, specialist, other), at a hospital; from the community; other)
- Other important aspects of the setting (social, cultural, economic environment, climate, other)
- Any information about the settings and locations that could have influenced the observed results (problems with transportation that might have affected patient participation or delays in administering interventions)
- Information not available_________________________________________

Example. **“** The study took place at the antiretroviral therapy clinic of Queen Elizabeth Central Hospital in Blantyre, Malawi. Blantyre is the major commercial city of Malawi, with a population of 1 000 000 and an estimated HIV prevalence of 27% in adults in 2004."

**Eligibility criteria for centers and those performing the interventions.**

Please describe the eligibility criteria:

- For centers:
  - center volume for the procedure of interest
  - other
- For care providers:
  - professional qualifications
  - years in practice
  - number of interventions performed
  - skill as assessed by level of complication
  - specific training before trial initiation
  - other
- Information not available_________________________________________

Example. “All participating centres… were major neurosurgical centres, treating large numbers of patients after aneurismal subarachnoid hemorrhage (SAH), each centre treating between 60 and 200 cases annually… Centres had to have expertise in both neurosurgical and endovascular management of ruptured aneurysms. Only accredited neurosurgeons with experience of aneurysm surgery were permitted to manage patients in the trial. Endovascular operators had to have done a minimum of 30 aneurysm treatment procedures, before they were permitted to treat patients in the trial.”

**Interventions (NPT rehab etc)**

**The interventions for each group with sufficient details to allow replication, including how and when they were actually administered**

Please provide a detailed explanation of the experimental intervention, including:

- - The type of the intervention (rehabilitation, behavioral treatment, education, psychotherapy or other)
  - The content of each session and the content of the information exchanged with participants
  - If the intervention was delivered to an individual or a group
  - Whether the treatment was supervised
  - Any instruments used to provide information (computers, tablets, smartphones, other)
  - The number and timing of sessions
  - The duration of each session, each main component of each session, and overall duration of the intervention
  - Any procedures for tailoring the interventions to individual participants (to patients’ comorbid conditions, tolerance, clinical course, other)
  - Any co-interventions permitted or restricted
- Information not available_________________________________

**Example**. "The exercise training program… consisted of 2 approximately 3-month-long phases of exercise training. The initial phase of exercise was designed to prepare the participants for progressive resistance training and also to minimize injury… Exercises during the first 3-month phase (phase 1) were conducted by a physical therapist using a group format (2-5 participants/group) and were designed to enhance flexibility, balance, coordination, movement speed, and, to some extent, strength of all major muscle groups. Twenty-two exercises formed the basis of this program (protocol available from the authors). The exercises were made progressively more difficult by increasing the number of repetitions and/or by performing the exercises in more challenging ways. The exercises were modified by the physical therapist to accommodate and/or target each participant's specific physical impairments. Common adaptations included increased time and/or attention to flexibility exercises targeted at hip flexors and extensors of the fractured leg, weight-shifting exercises, strengthening exercises for hip flexors, extensors, and abductors, and limitations of the amount of range of motion or weight used due to arthritis. Phase 1 exercises were progressed when the participant was able to perform the current level easily and the next level safely. When safely able, participants also exercised on a stationary bicycle or treadmill. Participants attempted this exercise for a minimum of 5 minutes and progressed to a maximum of 15 minutes. The treadmill speed or bicycle resistance was set at the highest comfortable setting that was safe for the participant. A formal aerobic exercise training protocol was not performed. Exercise sessions lasted 45 to 90 minutes (with breaks), depending on the participant's ability and tolerance, which increased over the course of phase 1. During the second exercise phase (phase 2), progressive resistance training was added. One-repetition maximum (1-RM) voluntary strength was measured on each of 6 different exercises (knee extension, knee flexion, seated bench press, seated row, leg press, and biceps curl), which were performed bilaterally on a Hoist weightlifting machine (Hoist Fitness Systems, San Diego, Calif). Initially, the participants performed 1 to 2 sets of 6 to 8 repetitions of each exercise at 65% of their 1-RM. By the end of the first month of weight training, they progressed to 3 sets of 8 to 12 repetitions performed at 85% to 100% of the initial 1-RM. The 1-RM measurements were repeated at 6 weeks and used to progressively increase each individual's exercise prescription. Participants continued to perform a shortened version of the phase 1 exercises and the treadmill or stationary bicycle warm-up exercise.”

Please provide a detailed explanation of the control intervention, including:

- - The type of control intervention (placebo, usual care, an active treatment, other)
  - If the control group is to receive “usual care”, describe thoroughly what that constitutes
  - If the control group or intervention group is to receive a combination of interventions:
    - provide a thorough description of each intervention,
    - present the order in which the combination of interventions were introduced or withdrawn, and if applicable the triggers for their introduction
  - Any other differences for the control intervention (detailed nature of the intervention, duration and frequency of attention, tailoring of the intervention, other)
  - If different from experimental intervention, any permitted or restricted co-interventions
- Information not available_____________________________________________

Example. “The [control] treatment follows the same format [as the experimental treatment], ie 10 weekly 90-min sessions. The therapist helps the patient identify daily stresses and discusses them in a supportive non-directive mode. No instructions for exposure are included. If the patient brings up trauma-related issues, the therapist gently redirects her to discuss other material.”

**Details of how the interventions were standardized.**

Please describe:

- Any standardization methods (training of care providers, equipment requirements, written manuals, specific guidelines, and materials used to train care providers to uniformly deliver the intervention)
- Where the reader may access materials used to standardize the intervention (by including a Web appendix with their article or a link to a stable Web site)
- Not applicable
- Information not available_____________________________________________

“The core content was standardized, although group leaders had flexibility in adapting the material and discussions to be relevant to the needs and characteristics of a given group. The Coordinating Center at UCSD provided continuing oversight for quality assurance (QA) for intervention sessions and related activities across sites. These activities included standardization of the intervention program by providing session materials, training and oversight of the group sessions across sites; structuring of overall content of group sessions; monitoring group sessions by logging attendance and a post-group summary; and teleconferencing with staff across sites at least once per month (weekly during the initial phase of the study) to promote uniformity of the delivery and responses to issues that arise.”

**Details of how adherence of care providers with the protocol was assessed or enhanced.**

Please describe:

- Any means to assess or enhance care provider adherence (review of case report forms, videotapes, audiotapes, other)
- Not applicable
- Information not available___________________________________________

Example. “All therapy sessions are videotaped… A senior clinician who is independent of… treatment delivery will rate 10% of the videotapes using measures adapted from several randomized clinical trials of psychotherapy…; the 10% figure was chosen arbitrarily in an attempt to ensure an adequate sample of information from each treatment condition.”

**Outcomes**

**Completely defined pre-specified primary and secondary outcome measures, including how and when they were assessed**

Please provide a detailed description of the primary outcome(s), by describing:

- - The variable of interest (ie pain, quality of life, clinical improvement, clinical event, other)
  - How the variable was defined (ie proportion of pain scores over 7, average quality of life score, change in blood pressure, number of myocardial infarctions, other)
  - How the variable was measured (ie visual analog scale, SF-36, systolic blood pressure, according to the WHO diagnostic criteria, other)
  - The overall time frame indicating pre-specified time point(s) (difference from baseline to 3 months; average over 1 year (at baseline, 6, and 12 months); final values at 1 month; time to event within 30 days; time to event or pre-specified final day of follow-up [indicate date]; other)
  - Who assessed the outcome (the patient, doctor, nurse, caretaker, other)
  - If any special skills or training were required for the outcome assessment
- Information not available_________________________________

**Example 1**. "The primary endpoint with respect to efficacy in psoriasis was the proportion of patients achieving a 75% improvement in psoriasis activity from baseline to 12 weeks as measured by the PASI [psoriasis area and severity index].”

Please provide a detailed description of the secondary outcome(s), by describing:

- - The variable of interest (ie pain, quality of life, clinical improvement, clinical event, other)
  - How the variable was defined (ie proportion of pain scores over 7, average quality of life score, change in blood pressure, number of myocardial infarctions, other)
  - How the variable was measured (ie visual analog scale, SF-36, systolic blood pressure, according to the WHO diagnostic criteria, other)
  - The overall time frame indicating pre-specified time point(s) (difference from baseline to 3 months; average over 1 year (at baseline, 6, and 12 months); final values at 1 month; time to event within 30 days; time to event or pre-specified final day of follow-up [indicate date]; other)
  - Who assessed the outcome (the patient, doctor, nurse, caretaker, other)
  - If any special skills or training were required for the outcome assessment
- Information not available_________________________________

**Example 2**. “Participants underwent a series of assessments at baseline and at 3 and 6 months after baseline using standardized procedures and forms. Follow-up data collection at 3 and 6 months was attempted for all participants, even those who had discontinued the therapy to which they had been assigned or who had received less than the full dose of exercise. Secondary outcomes: Participants also underwent assessments of muscle strength, gait, balance, body composition, and quality of life. Maximal voluntary muscle strength for knee extension and flexion of the fractured and unfractured limbs was measured using Cybex isokinetic dynamometry (Cybex International, Medway, Mass). Gait speed was measured for the middle 7 m of a 12-m walkway at the participant's self-selected and maximal gait speed. Balance was measured with the Progressive Romberg Test, Berg Balance Instrument, and a timed single-limb stance. Total body dual-energy x-ray absorptiometry (DEXA) (Hologic QDR1000/W, software version 6.2OD, Waltham, Mass) was used to assess total and regional fat-free mass and bone mineral density (BMD). The Medical Outcomes Study Short-Form 36 (SF-36) instrument and a modified version of the Hip Rating Questionnaire were administered to measure quality of life. The procedures for these measurements have been described in detail.  Except for DEXA measurement, the research staff who conducted the assessments were not involved in exercise training and were blinded to group assignment.”

**Any changes to trial outcomes after the trial commenced, with reasons**

Please describe any:

- Unplanned changes to: eligibility criteria, interventions, examinations, data collection, methods of analysis, and outcomes.
- Justification for unplanned changes (external evidence from other trials or systematic reviews suggesting the end point might not be appropriate, or recruitment or the overall event rate in the trial may be lower than expected, other)
- Not applicable
- Information not available_____________________________________________

Example. "The original primary endpoint was all-cause mortality, but, during a masked analysis, the data and safety monitoring board noted that overall mortality was lower than had been predicted and that the study could not be completed with the sample size and power originally planned. **The steering committee therefore decided to adopt co-primary endpoints of all-cause mortality (the original primary endpoint), together with all-cause mortality or cardiovascular hospital admissions (the first prespecified secondary endpoint)**."
