# Supplementary material for: Impact of an online writing aid tool for writing a randomized trial report: the COBWEB (Consort-based WEB tool) randomized controlled trial
Source: BMC Med. 2015 Sep 15;13:221. doi: 10.1186/s12916-015-0460-y (PMC4570037; doi:10.1186/s12916-015-0460-y)
Supplement: Additional file 3: — The CONSORT-based writing tool. (DOCX 19 kb) [file 12916_2015_460_MOESM3_ESM.docx]

**Additional file 3**

Example of the scoring system for completeness of reporting for the domain “Blinding”

| **Protocol id** | **Key element to report (Subitem)** | **Keyword or phrase** | **Keyword reported** | **Subitem**  **reporting**  **(0 to 1)** | **Subitem weight**  **(0-10)** | **Total score**  **(0-10)**  **(sum of subitem reporting*weights)** |
| --- | --- | --- | --- | --- | --- | --- |
| **x** | Blinding of study participants | Study participants were not blinded (or were aware of) treatment assignments | Yes | 1 | 2.5 | 7.25 |
|  | Blinding of care providers | Care providers were not blinded (or were aware of) treatment assignments | Yes | 1 | 2.5 |  |
|  | Blinding of those administering co-interventions | Those administering co-interventions were not blinded (or were aware of) treatment assignments | No | 0 | 0 |  |
|  | Blinding of outcome assessors | Primary outcome assessors were blinded to treatment assignments | No | 0.5 (1/2 keywords reported) | 2.5 |  |
|  |  | Secondary outcome assessors were not blinded to treatment | Yes |  |  |  |
|  | Blinding of data analysts | Data analysts were blinded to or unaware of treatment assignments | No | 0 | 0.5 |  |
|  | How was blinding maintained | All identification of treatment assignment was masked in medical files outcome assessors reviewed. | Yes | 1 | 0.5 |  |
|  | Similarity or differences of treatments | The interventional group received informational counseling on do not resuscitate options while the control group did not receive any counseling. | Yes | 1 | 0.5 |  |

Calculating total score (sum of subitem reporting*weights) = (1*2.5) + (1*2.5) + (0*0) + (0.5*2.5) + (0*0.5) + (1*0.5) + (1*0.5) = 7.25
