# Supplementary material for: Impact of an online writing aid tool for writing a randomized trial report: the COBWEB (Consort-based WEB tool) randomized controlled trial
Source: BMC Med. 2015 Sep 15;13:221. doi: 10.1186/s12916-015-0460-y (PMC4570037; doi:10.1186/s12916-015-0460-y)
Supplement: Additional file 4: — Weights for the scoring system for the completeness of reporting essential items according to intervention type. (DOCX 18 kb) [file 12916_2015_460_MOESM4_ESM.docx]

**Additional file 4**

Weights for the scoring system for the completeness of reporting essential items according to intervention type

Pharmacological therapy

| **Essential element to report** | **Weight** |
| --- | --- |
| TD: Type of trial design (parallel, etc) | 10 |
| Randomization: Sequence generation | 5 |
| Randomization: Allocation concealment | 5 |
| Blinding: participants | 5 |
| Blinding: outcome assessors | 5 |
| Participants: Inclusion and exclusion criteria | 10 |
| Intervention: Medication name | 2.5 |
| Intervention: Dose and duration of administration | 2.5 |
| Intervention: Number and timing of medication administration | 2.5 |
| Intervention: Name or type of control treatment | 2.5 |
| Outcomes: Presentation of primary outcome | 5 |
| Outcomes: Time frame for the primary outcome | 5 |

Nonpharmacological therapy, surgical procedures

| **Essential element to report** | **Weight** |
| --- | --- |
| TD: Type of trial design (parallel, etc) | 10 |
| Randomization: Sequence generation | 5 |
| Randomization: Allocation concealment | 5 |
| Blinding: participants | 5 |
| Blinding: outcome assessors | 5 |
| Participants: Inclusion and exclusion criteria | 5 |
| Participants: Eligibility criteria for centers, center volume if pertinent | 5 |
| Intervention: Type of the intervention | 2 |
| Intervention: Anesthesia management | 2 |
| Intervention: Preoperative care | 2 |
| Intervention: Postoperative care | 2 |
| Intervention: Presentation of the control intervention | 2 |
| Outcomes: Presentation of primary outcome | 5 |
| Outcomes: Time frame for the primary outcome | 5 |

Nonpharmacological therapy, other

| **Essential element to report** | **Weight** |
| --- | --- |
| TD: Type of trial design (parallel, etc) | 10 |
| Randomization: Sequence generation | 5 |
| Randomization: Allocation concealment | 5 |
| Blinding: participants | 5 |
| Blinding: outcome assessors | 5 |
| Participants: Inclusion and exclusion criteria | 5 |
| Participants: Eligibility criteria for centers, center volume if pertinent | 5 |
| Intervention: Type of the intervention | 2 |
| Intervention: The content of each session | 2 |
| Intervention: The number and timing of sessions | 2 |
| Intervention: Duration of sessions | 2 |
| Intervention: Presentation of the control intervention | 2 |
| Outcomes: Presentation of primary outcome | 5 |
| Outcomes: Time frame for the primary outcome | 5 |
