# Supplementary material for: Impact of an online writing aid tool for writing a randomized trial report: the COBWEB (Consort-based WEB tool) randomized controlled trial
Source: BMC Med. 2015 Sep 15;13:221. doi: 10.1186/s12916-015-0460-y (PMC4570037; doi:10.1186/s12916-015-0460-y)

**Additional file 5**

Sensitivity analyses

To investigate the influence of the choice of weights we used for each item when assigning a global score to each domain, we conducted a sensitivity analysis by varying the weights over their possible range for each domain.

We determined each possible combination of weights for each domain, under the constraint that each item would have a positive weight, the weights would sum up to 10 and weights would be multiples of 0.5. The number of possible weighting schemes varied from less than one thousand to several tens of thousands for each domain, according to the number of items.

For each domain, the results were reanalyzed when varying the weighting scheme of the domain’s item over the full range. The differences between the scores obtained with and without the writing tool were then computed with their 95% confidence interval, to detect weighting schemes which would have modified the conclusion.

Results showed that no domain specific weighting scheme modified the study’s conclusion, which can thus be considered as robust towards the choice of weights.

**Figure S1.** Mean difference (black points) and 95% confidence interval (gray) over all ranges of weighting schemes for each domain. The original analysis is represented in red.


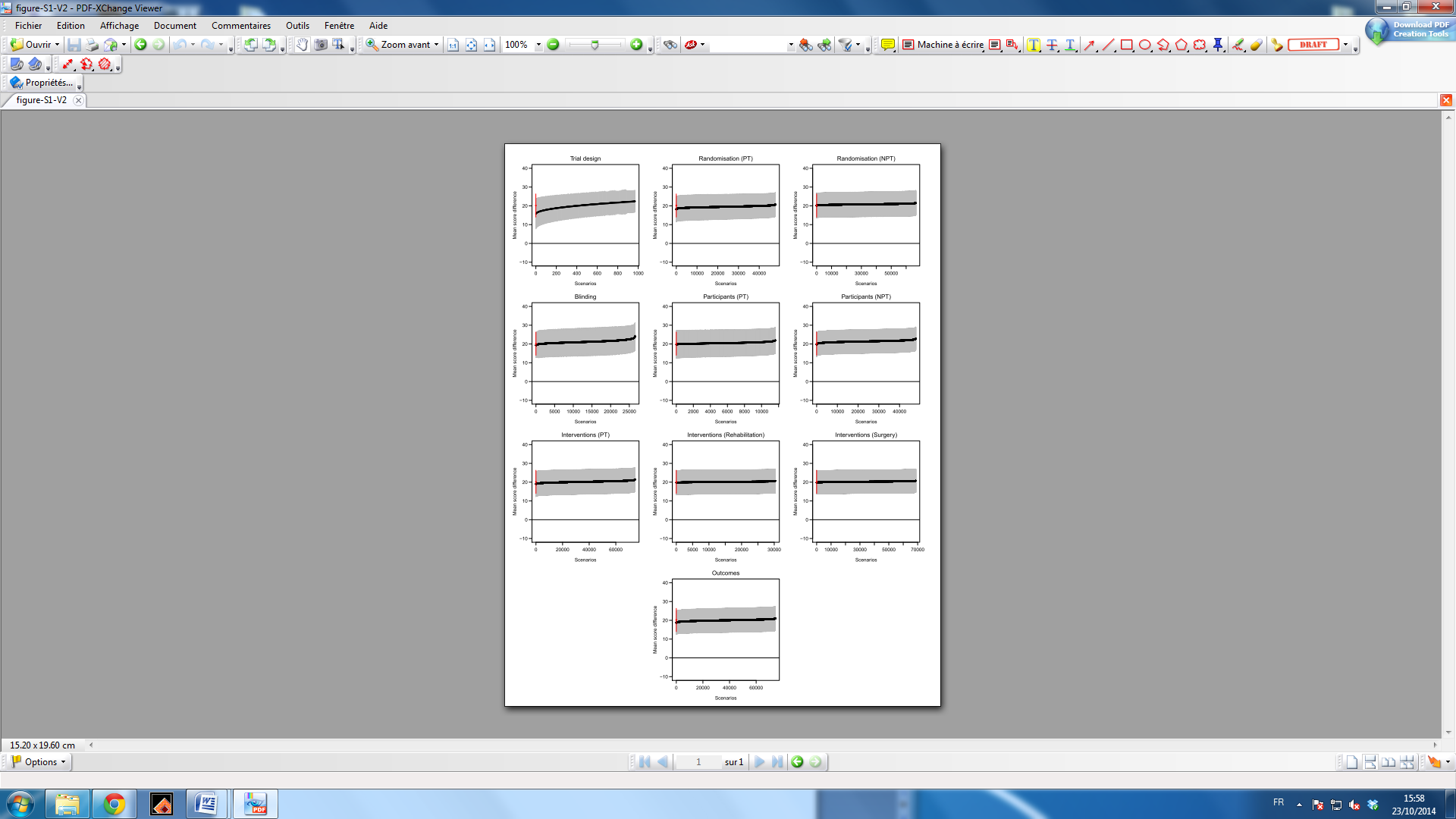

Supplement: Additional file 5: — Sensitivity analyses. (DOCX 257 kb) [file 12916_2015_460_MOESM5_ESM.docx]
